# Ultradeformable Archaeosomes for Needle Free Nanovaccination with *Leishmania braziliensis* Antigens

**DOI:** 10.1371/journal.pone.0150185

**Published:** 2016-03-02

**Authors:** Leticia H. Higa, Laura Arnal, Mónica Vermeulen, Ana Paula Perez, Priscila Schilrreff, Cecilia Mundiña-Weilenmann, Osvaldo Yantorno, María Elena Vela, María José Morilla, Eder Lilia Romero

**Affiliations:** 1 Nanomedicine Research Program, Departamento de Ciencia y Tecnologia, Universidad Nacional de Quilmes. Roque Saenz Peña 352, Bernal, Argentina B1876BXD; 2 Instituto de Investigaciones Fisicoquímicas Teóricas y Aplicadas (INIFTA), Universidad Nacional de La Plata-CONICET, Sucursal 4 Casilla de Correo 16, 1900 La Plata, Argentina; 3 Instituto de Estudios de la Inmunidad Humoral (IDEHU), CONICET-UBA, Facultad de Farmacia y Bioquímica, Universidad de Buenos Aires. Junin 956, 4° piso, 1113, Buenos Aires, Argentina; 4 Centro de Investigaciones Cardiovasculares, Universidad Nacional de La Plata, La Plata, Argentina; 5 Facultad de Ciencias Exactas, Centro de Investigación y Desarrollo de Fermentaciones Industriales (CINDEFI), UNLP. 50 No. 227, 1900 La Plata, Argentina; National Center for Cell Science, INDIA

## Abstract

Total antigens from *Leishmania braziliensis* promastigotes, solubilized with sodium cholate (ds*L*p), were formulated within ultradeformable nanovesicles (ds*L*p-ultradeformable archaeosomes, (ds*L*p-UDA), and ds*L*p-ultradeformable liposomes (ds*L*p-UDL)) and topically administered to Balb/c mice. Ultradeformable nanovesicles can penetrate the intact *stratum corneum* up to the viable epidermis, with no aid of classical permeation enhancers that can damage the barrier function of the skin. Briefly, 100 nm unilamellar ds*L*p-UDA (soybean phosphatidylcholine: *Halorubrum tebenquichense* total polar lipids (TPL): sodium cholate, 3:3:1 w:w) of -31.45 mV Z potential, containing 4.84 ± 0.53% w/w protein/lipid ds*L*p, 235 KPa Young modulus were prepared. *In vitro*, ds*L*p-UDA was extensively taken up by J774A1 and bone marrow derive cells, and the only that induced an immediate secretion of IL-6, IL-12p40 and TNF-α, followed by IL-1β, by J774A1 cells. Such extensive uptake is a key feature of UDA ascribed to the highly negatively charged archaeolipids of the TPL, which are recognized by a receptor specialized in uptake and not involved in downstream signaling. Despite ds*L*p alone was also immunostimulatory on J774A1 cells, applied twice a week on consecutive days along 7 weeks on Balb/c mice, it raised no measurable response unless associated to UDL or UDA. The highest systemic response, IgGa2 mediated, 1 log lower than im ds*L*p Al_2_O_3_, was elicited by ds*L*p-UDA. Such findings suggest that *in vivo*, UDL and UDA acted as penetration enhancers for ds*L*p, but only ds*L*p-UDA, owed to its pronounced uptake by APC, succeeded as topical adjuvants. The actual TPL composition, fully made of sn2,3 ether linked saturated archaeolipids, gives the UDA bilayer resistance against chemical, physical and enzymatic attacks that destroy ordinary phospholipids bilayers. Together, these properties make UDA a promising platform for topical drug targeted delivery and vaccination, that may be of help for countries with a deficient healthcare system.

## Introduction

Vaccination is considered the best hope for control of all forms of leishmania diseases, and the development of a safe, effective and affordable antileishmanial vaccine is a critical global public-health priority [1]. Nanovaccination, an approach relying on the higher uptake of nanoparticles compared to that of soluble material by antigen presenting cells (APC), results in enhanced antigen processing, lymphocyte priming and subsequent adaptive immune response. A growing number of *ad-hoc* designed nanoparticles is being tested as first (soluble or nanoparticle associated antigens without immunomodulatory activity) or second (nanoparticle associated antigens with immunomodulatory activity) generation adjuvants [2], entering clinical trials and commercialization [3]. The nanovaccination approach offers a new portfolio of adjuvants, which may help to overcome the challenges of vaccinating in countries of poor sanitary infrastructure, lacking of trained personnel, cold chain maintenance, proper waste management, syringes sterilization.

Probably because of its character of neglected disease, up to the moment no commercial vaccine is available to prevent the muco-cutaneous leishmaniasis (MCL), a highly morbid, inflammatory and disfiguring infection, mostly prevalent in Southamerica [4]. One of the factors contributing to slow down its development is the important genetic and biological divergence amongst *L*. *major /L*. *infantum* and *L*. *braziliensis* [5], which demands that polyvalent vaccines include a critical fragment of *L*. *braziliensis* antigens [6–8]. The recombinant antigens LEISH F1, is a protein comprised of three fragments conserved across various *Leishmania* species including *L*. *donovani*, and *L*. *chagasi*, causative agents of New World visceral leishmaniasis, and *L*. *braziliensis* [9, 10]. A vaccine in development by the Infectious Disease Research Institute (IDRI, Seattle, WA) and currently in phase I and II clinical trials combines LEISHF1 with the powerful adjuvants MPL-SE. The last consists of the TLR4 ligand Monophosphoryl Lipid A, an attenuated form of Lipid A from Salmonella Minnesota R595 (GSK), in a stable oil-in-water emulsion (made of squalene, Pluronic F68, glycerol, α-tocopherol and phospholipids). Squalene emulsions adjuvanted vaccines, although used against life threatening infections, have raised a number of concerns; are said for instance, to be excellent for priming, but not to boost pre existing immune responses well [11], other have been associated with narcolepsy in children and adolescents in northern European countries [12]. Emulsion adjuvants in general can bring also stability challenges for many antigens, particularly if a single dose liquid vial is preferred [13]. Whereas the overall risk benefit in prophylactic vaccination against pandemic and prepandemic lethal viral fevers was declared positive by the WHO [14], the use of squalene emulsions to adjuvant vaccines against a disease caused by a non lethal protozoan parasite is at least uncertain.

In this scenario, developing adjuvants different from squalene-containing o/w emulsions for vaccination against protozoan becomes an attractive subject to address. For instance, an initial study performed in our laboratory using for the first time archaeosomes, nanovesicles made of total polar lipids (TPL) from the hyperhalophile archaea *Halorubrum tebenquichense*, to immunize C3H/HeN mice against the model antigen bovine serum albumin, showed these archaeosomes recalled antigen specific Th1 biased and memory responses [15]. Later, we found that 3 sc doses of *Trypanosoma cruzi* (a protozoan trypanosomatide parasite close to *Leishmania*) total antigens adjuvanted with the same archaeosomes, were sufficient to protect Balb c mice from a lethal challenge with trypomastigotes of the *Tulahuen* strain [16]. These result encouraged us to test novel adjuvants containing TPL from *H*. *tebenquichense*, to elicit protective responses against protozoan parasites by non parenteral route. Topical and mucosal vaccines offer several advantages over injectables: are easier to administer, carry less risk of transmitting infections, and could simplify the manufacture process, thereby facilitating vaccine production and mass deployment [17]. Because of this, the present proof of concept is aimed to test the immune response by topical nanovaccination on Balb/c mice, to proteins extracted from *Leishmania braziliensis*. To that aim the proteins were loaded within nanovesicles having the same TPL than original archaeosomes, plus an edge activator that decreases the Young modulus of the bilayer, named ultradeformable archaeosomes (UDA). More specifically, the UDA are ultradeformable nanovesicles made of soybean phosphatidylcholine (SPC): sodium cholate (NaChol) and TPL at 3: 1: 3 w: w. The actual TPL composition, fully made of *sn* 2,3 ether linked saturated archaeolipids, gives the archaeosomes bilayer resistance against chemical, physical and enzymatic attacks that destroy ordinary phospholipids bilayers [18]. The same as archaeosomes, UDA display higher chemical and colloidal stability than the so called ultradeformable liposomes (UDL, nanovesicles made of SPC:NaChol 6:1 w:w), (unpublished results). The same as UDL, upon topically applied under non occlusive conditions, the UDA can penetrate the intact *stratum corneum* up to the viable epidermis, with no aid of classical permeation enhancers that can damage the barrier function of the skin. The TPL content of UDA is responsible for its much more pronounced capture by phagocytic /immature antigen presenting cells than UDL [19]. Such properties, together with higher physic-chemical and colloidal stability make UDA a promising platform for topical drug targeted delivery and adjuvancy, that may be of help for countries with a deficient healthcare system. To the best of our knowledge, this is the first report proposing a needle free vaccination strategy against CL from a New World leishmania specie.

## Materials and Methods

### Materials

Soybean phosphatidylcholine (SPC) (phospholipon 90 G, purity >90%) was a gift from Phospholipid/Natterman, Germany. Sodium cholate (NaChol), 1,2-Dimyristoyl-*sn*-glycero-3-phosphoethanolamine-N-(Lissamine™ rhodamine B sulfonyl) (Rh-PE), Sephadex G-75, 3-(4,5-dimethylthiazol-2-yl)-2,5-diphenyltetrazolium bromide (MTT), aluminiumnitrat nonahydrat (Al(NO_3_)_3_ x 9H_2_O), 2,2'-Azino-bis(3-Ethylbenzthiazoline-6-Sulfonic Acid) (ABTS), Lipopolysaccharides from *Escherichia coli* (LPS) and horseradish peroxidase conjugated anti-mouse IgA antibody were from Sigma-Aldrich (St Louis, MO, USA). Anti-mouse IgG1 and IgG2a were from Santa Cruz Biotechnology. Horseradish peroxidase conjugated anti-mouse IgG was from Chemicon International Millipore. Roswell Park Memorial Institute (RPMI) 1640 and modified Eagle’s medium (MEM) were from Gibco®, Life Technologies (New York, USA). Fetal calf serum (FCS), antibiotic/antimycotic solution (penicillin 10,000 IU/ml, streptomycin sulfate 10 mg/mL, amphotericin B 25 μg/ml), glutamine, and trypsin/ethylenediaminetetraacetic acid were from PAA Laboratories GmbH (Pasching, Austria). Complete RPMI (comp-RPMI) was prepared with RPMI 1640, 10% FCS, 5.5×10^−5^ M β-mercaptoethanol from Sigma-Aldrich, and antibiotic/antimycotic solution. Dendritic cell RPMI (DC-RPMI) was prepared with comp-RPMI plus 100 IU/ml of murine recombinant granulocyte-macrophage colony-stimulating factor (rmGM-CSF) (Pepro Tech, Rocky Hill, NJ, USA). All other chemicals and reagents were of analytical grade.

### Archaebacteria growth, extraction and characterization of total polar lipids

*Halorubrum tebenquichense* archaeas, isolated from soil samples of Salina Chica, Península de Valdés, Chubut, Argentina were grown in 8 L batch cultures in basal medium supplemented with yeast extract and glucose [15]. Cultures were monitored by absorbance at 660 nm and harvested in late stationary phase for storage as frozen cell pastes.

Total lipids were extracted from frozen and thawed biomass using the Bligh and Dyer method modified for extreme halophiles and the Total Polar Lipid (TPL) fraction was collected by precipitation from cold acetone [20]. Between 90 and 120 mg TPL were isolated from each culture batch. The reproducibility of each TPL extract´s composition was routinely screened by phosphate content [21] and electro spray ionization mass spectrometry (ESI-MS) as described in Higa et al. 2012 [19].

### Cells

#### Keratinocytes and macrophages

Human keratinocytes (HaCaT cells) were supplied by Dr. Salvatierra of Fundación Instituto Leloir (Buenos Aires, Argentina) and murine macrophages (J774A.1 cells) were supplied by Dr. Ugalde from Instituto de Investigaciones Biotecnológicas, Universidad de San Martin (Buenos Aires, Argentina). Cells were routinely cultured in MEM supplemented with 10% FCS, 1% antibiotic/antimycotic and 2 mM glutamine, at 37°C in 5% CO_2_ and 95% humidity.

#### Leishmania parasites

*Leishmania braziliensis* promastigotes (strain HOM/BR75/M2903) supplied by Dr. Fragueiro and Dr. Luna from Instituto Nacional de Parasitología Dr Mario Fatala Chaben were cultured at 26°C in Schneider culture medium supplemented with 10% FCS, 100 U/ml penicillin, 100 μg/ml streptomycin and 2 mM glutamine. Promastigotes were harvested from stationary phase cultures by centrifugation, washed three times with 10 mM Tris buffer plus 0.9% w/v NaCl, pH 7.4 (Tris buffer) and frozen at -20°C prior to use.

#### Bone marrow-derived dendritic cells (BMDCs)

Six-month old Balb/c mice were used as bone marrow donors. BMDCs were generated as described previously [22]. Briefly, after all muscle tissues were removed from the femurs, the bones were washed twice with phosphate-buffer saline (pH 7.4) (PBS) and transferred into a fresh dish with RPMI 1640 medium. Both ends of the bones were cut with scissors in the dish. Then the marrow was flushed out using 2 ml of RPMI 1640 with a syringe and 25-gauge needle. The tissue was suspended and passed through a 100 μm cell strainer (BD Falcon, Franklin Lakes, NJ, USA) to remove small pieces of bone and debris. Red cells were lysed with 0.45 M ammonium chloride, and the remaining cells were washed, and suspended at a concentration of 1 × 10^6^ cells/ml in DC-RPMI medium with two additional supplementation of medium containing rmGM-CSF in 100 mm Petri dishes (5 x10^6^ cells/dish). After 9 days of culture, approximately 80% of the harvested cells expressed MHC class II and CD11c.

### Preparation of detergent-solubilized *L*. *braziliensis* proteins

A whole-cell extract was prepared as described by Santos et al., 2006. Briefly, stationary phase promastigotes were washed three times in Tris buffer (5 min centrifuged at 250 g) disrupted by 10 freeze and thaw cycles, and submitted to probe-type ultrasonication (Sonics Vibra cell) at 50% amplitude (130 Watts), for 10 min in an ice bath. The protein concentration in the whole-cell extract was estimated by Bradford using ovalbumin as standard [23].

To obtain the detergent-solubilized *L*. *braziliensis* proteins (ds*L*p), the whole-cell extract of *L*. *braziliensis* was incubated with 10, 5 or 1.25% w/v NaChol for 45 min at 4°C; the resultant ds*L*p was recovered from filtration through 0.45 μm pores nylon filter.

Whole-cell extract and ds*L*p were run by electrophoresis in 7.5–15% linear gradient sodium dodecyl sulfate-polyacrylamide gels (SDS-PAGE) [24]. Briefly, the samples were mixed with 62.5 mM Tris-HCl pH 6.8; 2% w/v SDS, 10% v/v glycerol, 5% v/v β-mercaptoethanol and 0.001% w/v bromophenol blue and heated at 95°C for 5 min prior electrophoresis. Proteins were separated at a constant voltage of 100 V, using a running buffer containing 0.025 M Tris, 0.192 M glycine pH 8.3 and 0.1% w/v SDS. The staining was performed with Coomassie Brilliant Blue R-250 followed by silver staining. The ImageJ software (National Institutes of Health, Bethesda, MD) was used to scan the 63 kDa band of the ds*L*p, and to quantify the amount of ds*L*p associated to UDA or UDL. To that aim, a plot of the signal of growing amounts of ds*L*p protein vs mass protein (5–30 μg) was fitted by linear regression. Then the ds*L*p signal in UDA or UDL was extrapolated to render the actual protein mass associated to each nanovesicle.

### Preparation and characterization of nanovesicles

#### Preparation

Empty nanovesicles: ultradeformable archaeosomes (UDA, made of TPL:SPC:NaChol, 3:3:1 w:w), ultradeformable liposomes (UDL, made of SPC:NaChol, 6:1 w:w) and conventional liposomes (L, fully made of SPC); and ds*L*p-containing nanovesicles: ds*L*p-containing UDA (ds*L*p-UDA) and ds*L*p-containing UDL (ds*L*p-UDL), were prepared by the thin film hydration method.

Empty nanovesicles: appropriate amounts of SPC in chloroform, and TPL and NaChol in chloroform: methanol (1:1, v/v), were mixed in round bottom flasks. Solvents were rotary evaporated at 40°C until elimination, the lipid films were flushed with N_2_ and hydrated with aqueous phase (Tris-HCl buffer) up to a final concentration of 43 mg of phospholipids/ml followed by extrusion.

ds*L*p-containing nanovesicles: the lipid films were prepared as detailed above, but hydrated with ds*L*p having NaChol at 10, 5 and 1.25% w/v in Tris-HCl buffer) up to 43 mg of phospholipids/ml. The resultant suspensions were sonicated (45 minutes with a bath-type sonicator 80 W, 40 KHz) and extruded 15 times through three stacked 0.2–0.1- and 0.1-μm pore size polycarbonate filters using a 100 ml Thermobarrel extruder (Northern Lipids, Vancouver, Canada). After extrusion, nanovesicles were submitted to five freeze-thaw cycles between −70°C and 40°C. Finally, nanovesicles were separated from free ds*L*p by gel filtration on Sephadex G-75 using the minicolumn centrifugation technique [25].

Rh-PE-labelled vesicles: Rh-PE was dissolved in chloroform and 12.5 nmol was added to the organic solution of lipids (nearly 2800:1 w:w phospholipids:Rh-PE); nanovesicles were prepared as detailed above.

#### Quantification

Phospholipids were quantified by Bötcher microassay [21]. The ds*L*p proteins associated to UDA or UDL was quantified as stated in *Preparation of detergent-solubilized L*. *braziliensis proteins*.

#### Size and Z potential

Size and Zeta potential were determined by dynamic light scattering (DLS) and phase analysis light scattering (PALS) respectively, using a nanoZsizer apparatus (Malvern Instruments, Malvern, United Kingdom).

#### Morphology

Aliquots of nanovesicles were dropped on a standard carbon-coated cooper transmission electron microscopy (TEM) grid and then air-dried at room temperature overnight. TEM images were obtained with a JEM 1011 (Jeol, New York, NY) electron microscope at 80 kV.

Aliquots of nanovesicles diluted in Tris buffer with 10 mM CaCl_2_ were dropped on mica as the substrate. Vesicles were stood for 15 min to be washed with 1 ml of Milli-Q water. Atomic force microscopy (AFM) images were obtained with a Multimode Scanning Probe Microscope (Veeco, Santa Barbara, CA) equipped with a Nanoscope V controller operating in tapping mode at room temperature.

#### Deformability

The nanovesicles deformability (*D*) was calculated according to Van den Bergh [26], having *D = J(rv/rp)*^*2*^, where J is the rate of penetration through a permeability barrier, rv is the size of nanovesicles after extrusion, and rp is the pore size of the barrier. To measure J, nanovesicles were extruded through two stacked 50 nm (rp) membranes at 0.8 MPa using a Thermobarrel extruder. Extruded volume was collected along 15 min, each fraction was quantified for phospholipids, and J calculated as the area under the curve of the plot of recovered phospholipids as a function of time. The average vesicle diameter after extrusion (rv) was measured by DLS.

### Young Modulus determination through Atomic Force Microscopy (AFM)

#### Sample preparation and Force Curves acquisition

The mechanical properties of conventional, non ultradeformable (SPC), empty (UDA, UDL) and ds*L*p nanovesicles were determined as described by Arnal et al. 2012 [27]. All the measurements were done using a Multimode Scanning Probe Microscope (Veeco, Santa Barbara, CA) equipped with a Nanoscope V controller. The nanovesicles were electrostatically immobilized onto polyethyleneimine (0.1% w/v) pre-incubated glass slides and immediately mounted in the AFM liquid chamber. Fifty microliters of PBS were added to the chamber in order to maintain the samples hydrated during the course of the experiments. The measurements were done using contact sharpened silicon nitride probes (NP-10, Veeco) with a nominal tip radius between 20–60 nm. The cantilever spring constant (K_c_) was determined for every probe before starting the measurements using the Thermal Tune method and flat muscovite mica (SPI V-1 grade) as a flat rigid surface for photodetector sensitivity calibration, the experimental K_c_ values were between 0.08 and 0.13 N/m. To acquire the Force vs. Distances curves on the surface of nanovesicles the Force Volume (FV) tool with a routine of 32 x 32 force curves at a scan rate of 1Hz was used. Note that different scan rates within 0.1 and 2 produced similar results. A limit in the highest applied force (10 nN) was established in order not to damage the samples during the FV acquisition.

#### Force curves analysis and Young Modulus determination

The nanoindentation analysis was done using in house developed software [27] following previously described procedures [28–30]. First we determined the contact point of the force curves; which was done manually by determining the exact point were the curves begin to lift from the noncontact baseline. Then the approximation segment of the Force vs. Distance curves were transformed into Force vs. Indentation curves by subtracting at constant loading force the Z-displacement measured in a vesicle and the respective Z-displacement measured on a hard surface (clean flat mica). All the resulting Force vs. Indentation curves showed a non-linear behaviour at low force, which were fitted using a Hertz model to determine the Young Modulus. The selected Hertz model was the one that considers a conical tip shape since it was the model that bests fitted our experimental data. The equation for the Hertz model [31, 32] is the following: F = 2E tanα δ^2^/π (1-ν^2^); where F is force, E is the Young Modulus, α is the half opening angle of the conical indenter (53°; based in geometrical characteristics of the tip and scanning electron microscopy observations), δ is the indentation and ν is the Poisson radius which for soft biological samples is assumed to be 0.5 [33, 34].

### Viability of keratinocytes, macrophages and bone marrow-derived dendritic cells

HaCaT, J774A.1 cells and BMDCs were seeded at a density of 5 x 10^4^ and 9 x 10^4^ cells per well, respectively, onto 96-well flat-bottom plates and grown for 24 h at 37°C. Then, the medium was replaced by 100 μL of fresh medium with 5% FCS containing decreasing concentrations of empty UDA/UDL or ds*L*p- UDA/UDL in a half-fold dilution series (1.6 to 0.2 mg/ml of phospholipids, corresponding to 72 to 9 μg/ml of ds*L*p in UDL and 56 to 7 μg/ml of ds*L*p in UDA) or ds*L*p alone (200 to 50 μg/ml) and cells were incubated at 37°C for 24 h. After that, the medium was removed and replaced by 0.5 mg/ml of MTT. After 3 hours of incubation, the MTT solution was removed, the insoluble formazan crystals were dissolved in dimethyl sulfoxide, and absorbance was measured at 570 nm in a microplate reader (Dynex Technologies, MRX tc, Chantilly, Virginia). The cell viability was expressed as a percentage of the viability of cells grown in medium.

### Pro-inflammatory cytokine production

J774A.1 and BMDCs were seeded at a density of 5 x 10^4^ cells per well onto 24-well plates and grown for 24 h at 37°C. Then, the medium was replaced by fresh medium with 5% FCS containing: empty UDA and UDL, ds*L*p-UDA and ds*L*p-UDL (0.8 mg/ml phospholipids), ds*L*p alone (10 and 50 μg/ml) and LPS (1 μg/ml). Supernatants were collected at 14 h and 48 h and TNF-α, IL12p40, IL 6 and IL1-β production were measured by ELISA using a BD Kit.

### Nanovesicles uptake by BMDC

The uptake of Rh-PE-labeled nanovesicles by BMDCs was determined by flow cytometry. BMCDs cells seeded at a density of 3.5×10^5^ cells per well onto 6-well microplates were grown for 24 h at 37°C. The medium was replaced with fresh RPMI with 5% FCS containing Rh-PE-labeled nanovesicles, UDA and UDL at 0.8 mg/ml phospholipids, and cells were incubated for 1, 3, and 5 h at 37°C. After incubation, the supernatant was removed and loose cells were washed with PBS. Cells were suspended in PBS, and a total of 1 x 10^5^ cells were analyzed by flow cytometry (BD FACSCalibur™; BD Biosciences, San Jose, CA, USA). Data were analyzed using WinMDI 2.9 software (Microsoft, Redmond, WA, USA).

### Immunization

Male 6-8-week-old Balb/c mice were obtained from Facultad de Ciencias Veterinarias, Universidad Nacional de La Plata. Mice were housed 5 per cage and keep in a ventilated room under controlled conditions at constant room temperature 22°C, with 12/12 h light-dark cycle and free access to food and water. All procedures requiring animals were performed in agreement with institutional guidelines and were approved by the Committee on the Ethics of Animal Experiments of the National University of Quilmes, Argentina. All animals were treated in a humance way, following the guidelines listed in “Guide for the Humance Care and Use of Laboratory Animals” (NIH publication). Five mice per group were immunized according to the schemes shown in Table 1. Topical samples were dropped on manually trimmed hair, intact back skin surface of each mouse over an area of 2 cm^2^. Mice were kept in individual cages for 30 min until drops had dried. At the end of experimentation (3 months) animals were euthanized by cervical dislocation.

**Table 1:** Immunization scheme.

Route of	Sample	Dose (μg ds*L*p/	Days of immunization
administration	50 μl	μg phospholipid	
Intramuscular	ds*L*p-Al_2_O_3_	36/-	0, 21
Topical	ds*L*p	36/-	Once a week for 7 weeks
Topical	ds*L*p-UDA	43/1205	Twice a week on consecutive days for 7 weeks
Topical	ds*L*p-UDL	50/1115	Twice a week on consecutive days for 7 weeks

ds*L*p-UDA, detergent-solubilized *L*. *braziliensis* proteins-containing- ultradeformable archaeosomes; ds*L*p-UDL, detergent-solubilized *L*. *braziliensis* proteins containing ultradeformable liposomes

Blood was collected from the tail veins at weekly intervals up to 8 weeks. Salivary washes were collected at 0, 35 and 49 days. To that aim, 100 μl of pilocarpine-HCl (1mg/ml in PBS) were i.p injected and after 1 min, the saliva was collected with a micropipette without damaging the gingival tissue. The samples were kept frozen at -20°C until use.

IgG antibody and isotypes in blood samples and IgA antibody in salivary washes samples were analyzed by ELISA. Briefly, microtiter plates were coated overnight at 4°C with 45 μg/ml ds*L*p in 0.1 M carbonate-bicarbonate buffer (pH 9.6) and then blocked with PBS containing 0.2% Tween 20 (0.2% PBST) for 1 h at 37°C after washing with 0.05% PBST. After another washing, 100 μL of three-fold dilutions of individual sera in 0.05% PBST was added. After 2 h at 37°C and further washing, the plates were incubated for 1 h at 37°C with horseradish peroxidase-conjugated goat anti-mouse IgG diluted 1:2000 in 0.025% PBST. To determine the antibody isotyping, horseradish peroxidase-conjugated rat anti-mouse IgG1 or IgG2a revealing antisera, diluted 1:1000, were used. The plates were further washed and incubated with ABTS for 10 min at room temperature in the dark. The absorbance was measured at 405 nm using a microplate reader. Antibody titers were represented as end-point dilutions exhibiting an optical density of 0.3 units above background.

The IgA titers of salivary washes were determined in the same fashion.

### Statistical analysis

Statistical analyses were performed by one-way analysis of variance followed by Turkey’s test sing Prisma 4.0 Software (Graph Pad, San Diego, California). Significance levels are shown in figure legends.

## Results

### Characterization of nanovesicles containing detergent-solubilized leishmania proteins

Both the whole-cell extract of leishmania promastigotes and its supernatant have been indistinctly used as antigenic material in *in vitro* tests and as pre-clinical and clinical vaccine candidates [35–37]. Besides, it has been observed that detergent-solubilized proteins of a crude extract of *L*. *amazonensis* amastigotes reconstituted in dipalmitoylphosphatidylcholine: dipalmitoylphosphatidylserine: cholesterol nanovesicles, produce protein specific antibodies and partially protects Balb/c mice to infection with *L*. *amazonensis* promastigotes [38]. Moreover, the centrifugation pellet of the whole-cell extract from *L*. *amazonensis* and *L*. *braziliensis* promastigotes was reported to be more antigenic than the supernatant [37].

**Fig 1:**
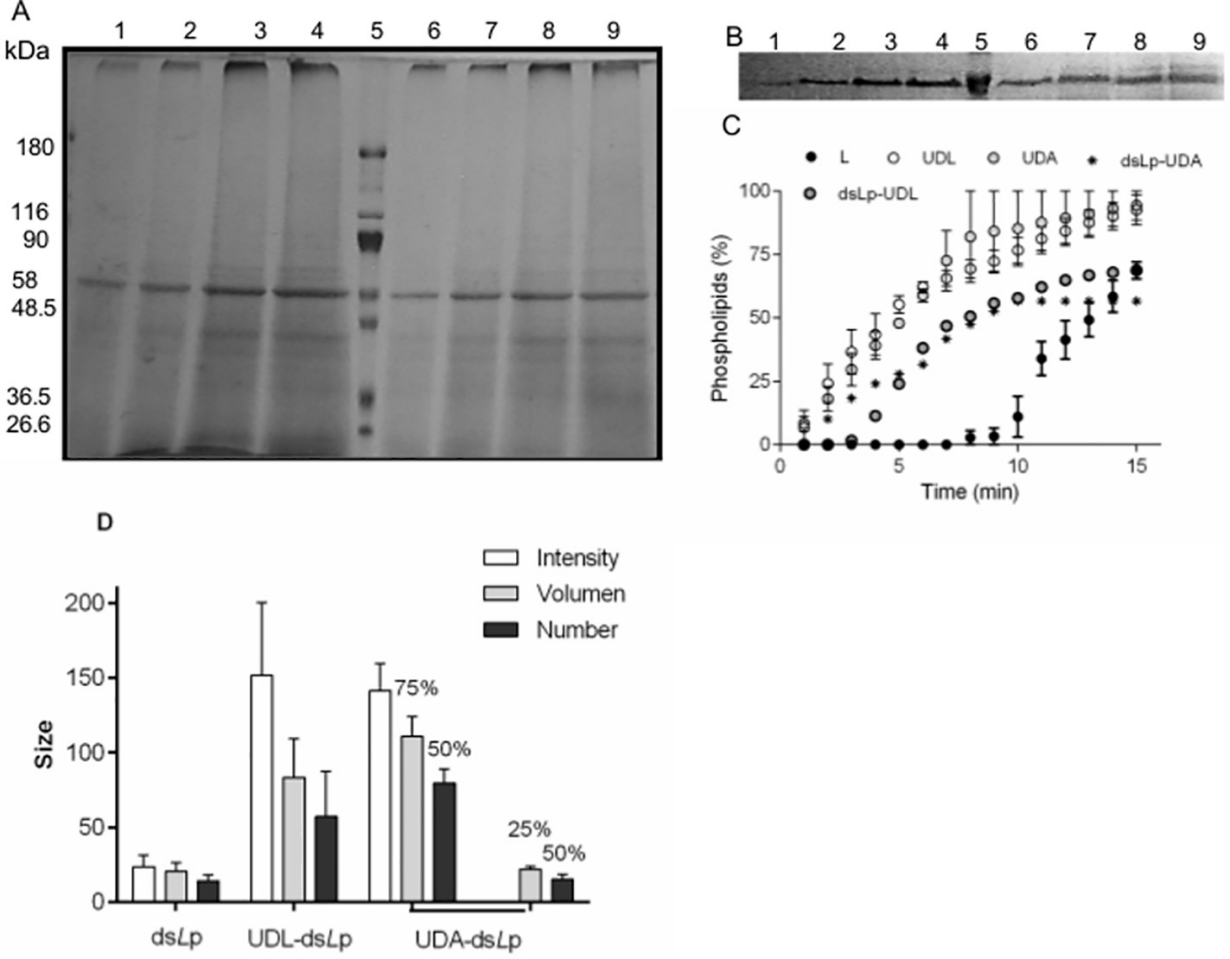
Characterization of nanovesicles. (A) Gradient (7.5%-15%) PAGE of whole-cell extract (5 to 30 μg, lanes 1 to 4) and ds*L*p (5 to 30 μg, lanes 6 to 9) from *L*. *braziliensis*, separated by molecular mass markers (lane 5). (B) Calibration curve PAGE for ds*L*p (5–30 μg, lanes 1 to 4); molecular mass marker (lane 5); ds*L*p-UDA (20 μl, lanes 6 and 7); ds*L*p-UDL (20 μl, lanes 8 and 9). (C) A plot of phospholipids from nanovesicles extruded across 50 nm pore size membranes versus time. Values represented mean ± SD. (D) Hydrodynamic diameter of ds*L*p and ds*L*p- nanovesicles measured by dynamic light scattering expressed in intensity, volume and number mode. The percentages indicated the proportion of each structure.

Formulating voluminous antigens higher than small model proteins within UD nanovesicles however, proved complex. For instance, attempts to solubilize the lipid film with whole-cell extract-containing buffer (or its centrifugation pellet) led to macroscopic precipitate (data not shown). To overcome such drawback, the whole cell extract was solubilized as smaller mixed micelles, which could be fully suspended in aqueous media, making feasible their trapping within the inner space of nanovesicles. The whole-cell extract of leishmania promastigotes can be solubilized employing different hydrophilic/hydrophobic balance and critical micellar concentration (cmc) detergents, such as sodium deoxycholate, NaChol, sodium dodecylsulphate or octylglucopiranoside [38–41]. We chose NaChol because it forms part of UDL and UDA bilayers as the edge activator and because of its non denaturing character. The cmc of NaChol is 0.6–0.7% w/v; above that limit, micelles of 14 nm size are formed; at 1% w/v, NaChol is known to solubilize more than 90% axonal membrane proteins of bovine brain [42]. We found that 1.25% w/v NaChol solubilized the whole-cell extract as 25 nm mixed micelles (ds*L*p) of 4.8 mg/ml proteins and 3.3 mg/ml phosphate. The SDS-PAGE of the whole-cell extract and of ds*L*p showed a similar band pattern (Fig 1A and 1B) (meaning that most of the proteins were solubilized within mixed micelles), dominated by a highly intense 63 KDa band accompanied by heavy proteins and less intense lighter bands. The 63 KDa band could correspond to the GP65 promastigote surface glycoprotein, already described in several species of leishmania [43, 44]. Since the presence of NaChol interferes with protein colorimeric methods, the densitometric intensity of the 63 KDa band was used to estimate the minimal protein content in ds*L*p and in nanovesicles. Structural properties of empty and ds*L*p-loaded nanonanovesicles are shown in Table 2.

**Table 2:** Structural features of nanovesicles.

Samples	Mean size(nm)(polydispersity index)	Z potential(mV)	Protein concentration(mg/ml)	Protein/ Phospholipid ratio(% w/w)	*D*	E (kPa)
L	109 ± 5(0.103)	-10 ± 0.3	-	-	765	1119 ± 242
UDL	110 ± 2(0.242)	-12 ± 2	-	-	3882	430 ± 137
UDA	130 ± 1(0.241)	-35 ± 4	-	-	4064	294 ± 177
ds*L*p	25 ± 3 (0.32)	-19 ± 3.6	5	-	-	-
ds*L*p-UDL	99.86 ± 8.33(0.19)	-11 ± 3.1	1.00 ± 0.60	4.84 ± 0.53	1100	161 ± 73
ds*L*p-UDA	103.75 ± 1.85(0.21)	-31 ± 3.7	0.85 ± 0.35	3.52 ± 0.25	1100	235 ± 69

Values represent means ± standard deviation (SD) (n = 8).

**Fig 2:**
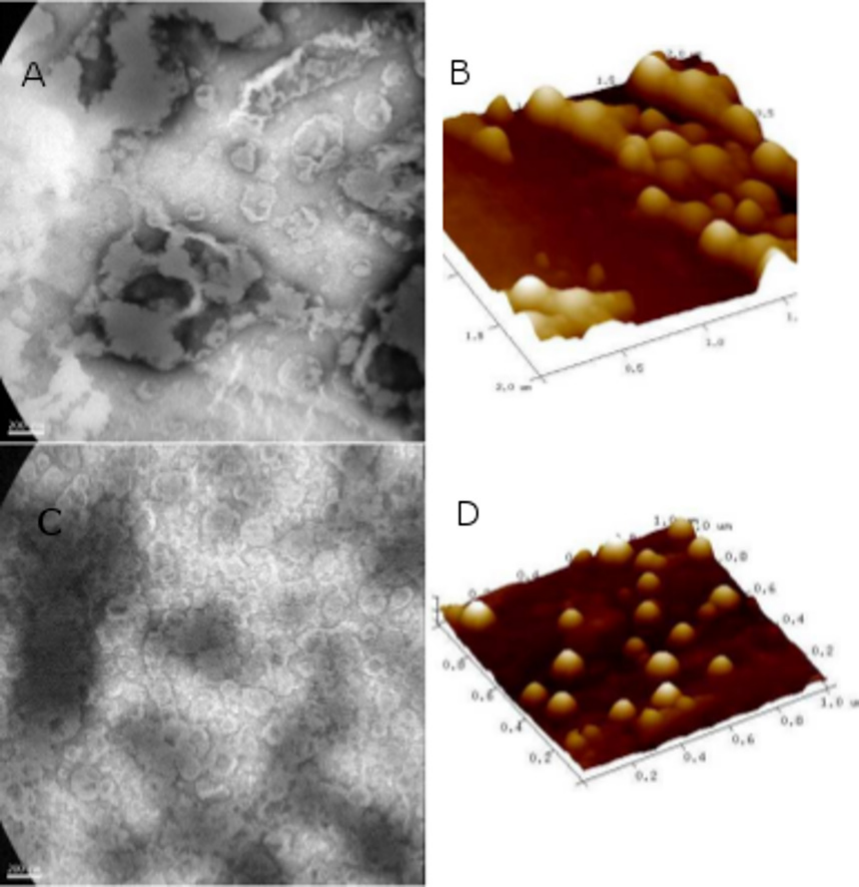
Morphology of nanovesicles. (A and B) ds*L*p-UDA. (C and D) ds*L*p-UDL. (A and C) TEM images (30000 X). (B and D) Three-dimensional AFM scans.

*D*, deformability; E, Young´s modulus; ds*L*p, detergent-solubilized *L*. *braziliensis* proteins; L, conventional liposomes; UDL, ultradeformable liposomes; UDA, ultradeformable archaeosomes; ds*L*p-UDA, detergent-solubilized *L*. *braziliensis* proteins containing ultradeformable archaeosomes; ds*L*p-UDL, detergent-solubilized *L*. *braziliensis* proteins containing ultradeformable liposomes.

ds*L*p-UDA and ds*L*p-UDL resulted to be ∼100 nm diameter nanovesicles, the same size order of UDA and UDL. The potential structural destabilization on nanovesicles induced by ds*L*p, was assessed by determining their size by DLS after 24 h; the presence of unilamellar nanovesicles was further confirmed by TEM (Fig 2A and 2C) and by AFM (Fig 2B and 2D). By DLS, the occurrence of heterogeneous populations having 15–25 nm diameter micelles growing at expenses of higher sized nanovesicles in particular for ds*L*p-UDA, was revealed. The size heterogeneity of nanovesicles was less pronounced for films suspended with ds*L*p 1.25% w/v NaChol (Fig 1D); for that reason, ds*L*p-nanovesicles were prepared with ds*L*p 1.25% w/v NaChol. By AFM the ds*L*p-UDA–despite of its high Z potential-, appeared as aggregated nanovesicles, probably because of ionic bridging with CaCl_2_ employed to fix the nanovesicles to the mica substrate.

The plot of phospholipids crossing a nanoporous barrier as a function of time is shown in Fig 1C. The calculated *D* of ds*L*p-nanovesicles was reduced nearly 3.5 folds as compared to empty nanovesicles, and nearly four folds above that of conventional non ultradeformable nanovesicles (Table 2).

### Young Modulus determination through AFM

The Young´s modulus (E) or longitudinal elastic modulus is the ratio between the increased tension applied by traction (in the zone of elastic behavior of the material) (dσ) and the resultant increased relative deformation (dε) (E: dσ/dε). E is a measure of a material rigidity: the higher the elastic modulus is the more rigid material.

AFM is a growingly used technique to acquire soft matter images, such as molecular crystals, proteins and live cells [45]. AFM is a well-suited technique for studying structural features of nanovesicles enabling the simultaneous nanoscale analysis of shape and mechanical properties of the bilayers. It allows picking up surface topographical images with a space resolution close to 1 Å and force vs. distance curves with a detection limit close to 10^−12^ N.

Our results showed in first place that E values for conventional nanovesicles are coincident with bibliographic data (1.97 ± 0.75 x 10^6^ Pa for adsorbed EggPC nanovesicles [46]. The Young´s modulus for UDL and UDA were between 3 and 4 folds lower than that of conventional nanovesicles. The difference was coincident with the higher *D* of UDA and UDL as compared to conventional non ultradeformable nanovesicles determined by the Van der Berg method. The Young´s module for ds*L*p- nanovesicles was in the order of the corresponding to empty ultradeformable nanovesicles (Table 2).

### Cell viability

**Fig 3:**
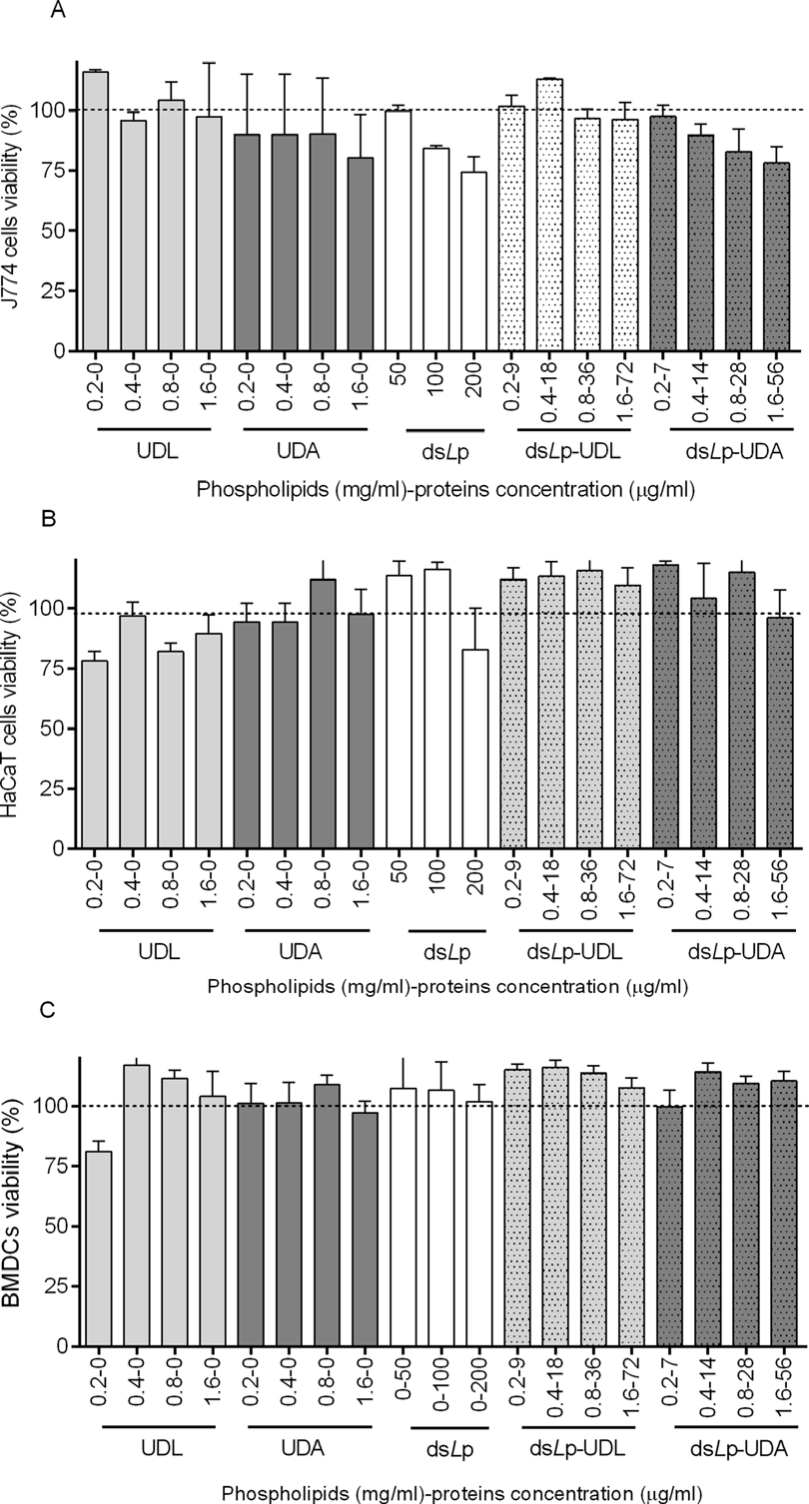
Cytotoxicity of empty and ds*L*p- nanovesicles. (A) J774 cells. (B) HaCaT cells. (C) Bone marrow derived dendritic cells (BMDC). Values represent mean ± SD (n = 5). Not significant differences were found between treatments and control cells.

Neither the empty or ds*L*p- nanovesicles nor ds*L*p in the range of tested concentrations were cytotoxic to macrophages, keratinocytes or BMDC (Fig 3).

### Pro-inflammatory cytokines induction on BMDC and J774A1 cells and uptake by BMDC

**Fig 4:**
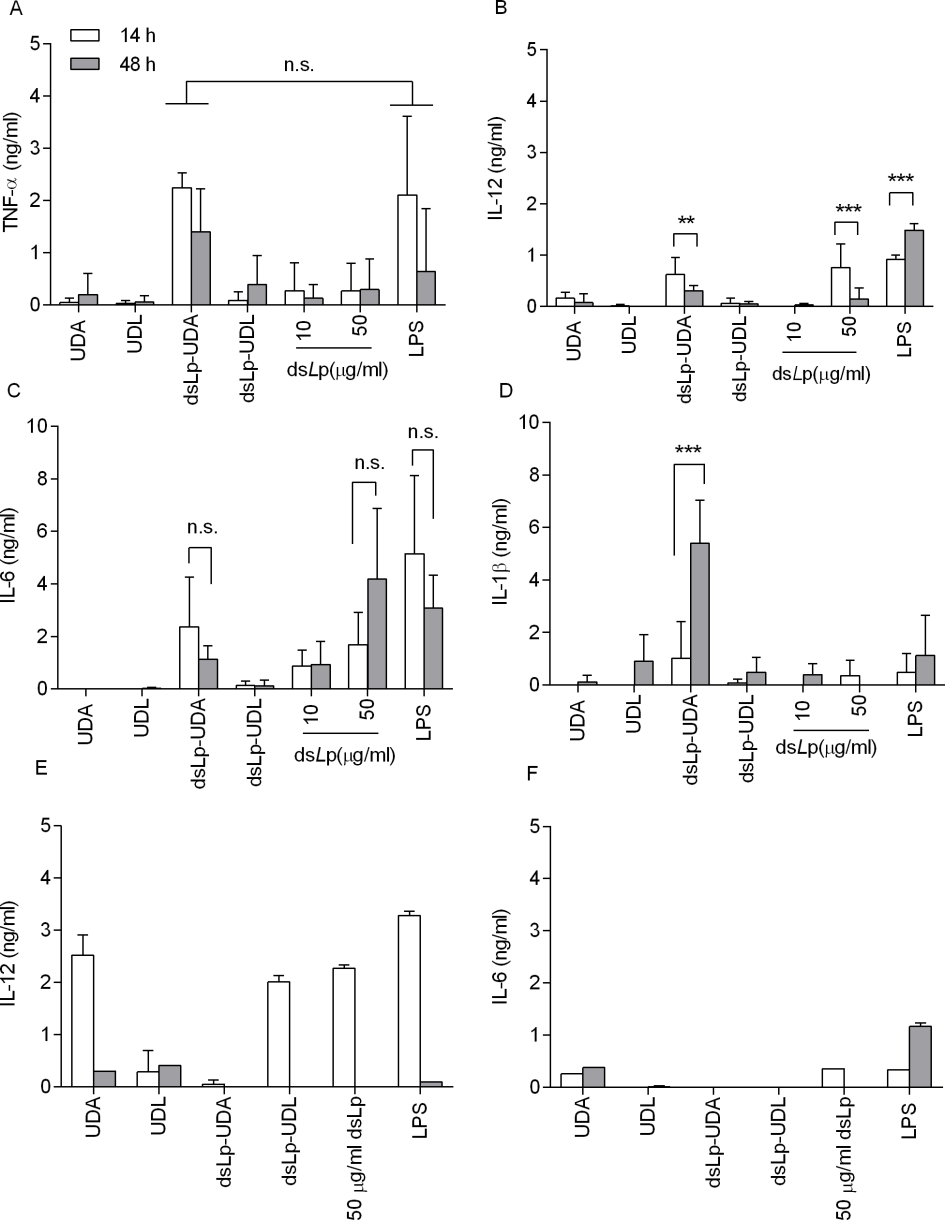
Cytokine levels in J774A1 macrophages and BMDC supernatants. **Cells were incubated along 14 and 48 h with UDA, UDL, ds*L*p-UDA, ds*L*p-UDL, ds*L*p alone and LPS (TLR4 agonist) as positive control.** The absorbance of the basal condition (non-stimulated cells, incubated with culture media as negative control) was subtracted to that of each cytokine concentration. Each point represents the media of n = 3 and its corresponding SD. ** denotes p < 0.01, *** denotes p < 0.001; n.s. not significant. (A) TNF- α levels in J774A1 supernatants. (B) IL-12p40 levels in J774A1 supernatants. (C) IL-6 levels in J774A1 supernatants. (D) IL-1β levels in J774A1 supernatants. (E) IL-12p40 levels in BMDC supernatants. (F) IL-6 levels in BMDC supernatants.

In (Fig 4A–4D) the cytokine levels on J774A1 macrophages supernatants induced by ds*L*p and ds*L*p-nanovesicles are depicted. Neither UDL, UDA nor ds*L*p-UDL induced pro inflammatory cytokines. ds*L*p alone, at 50 μg/ml induced an early onset of IL-12p40 followed by high level of IL-6; no TNF-α or IL-1 β induction was registered. ds*L*p-UDA however, was the only to induce an early onset of IL-6, IL-12p40 and TNF- α, followed 34 h later by high IL-1 β titers.

The cytokine response of BMDC to ds*L*p-UDA however, was milder and transient than in J774A1cells: no IL1-β or TNF-α were detected, low and transient titers of IL-6 and IL-12p40, this last fading after 48h (Fig 4E and 4F).

**Fig 5:**
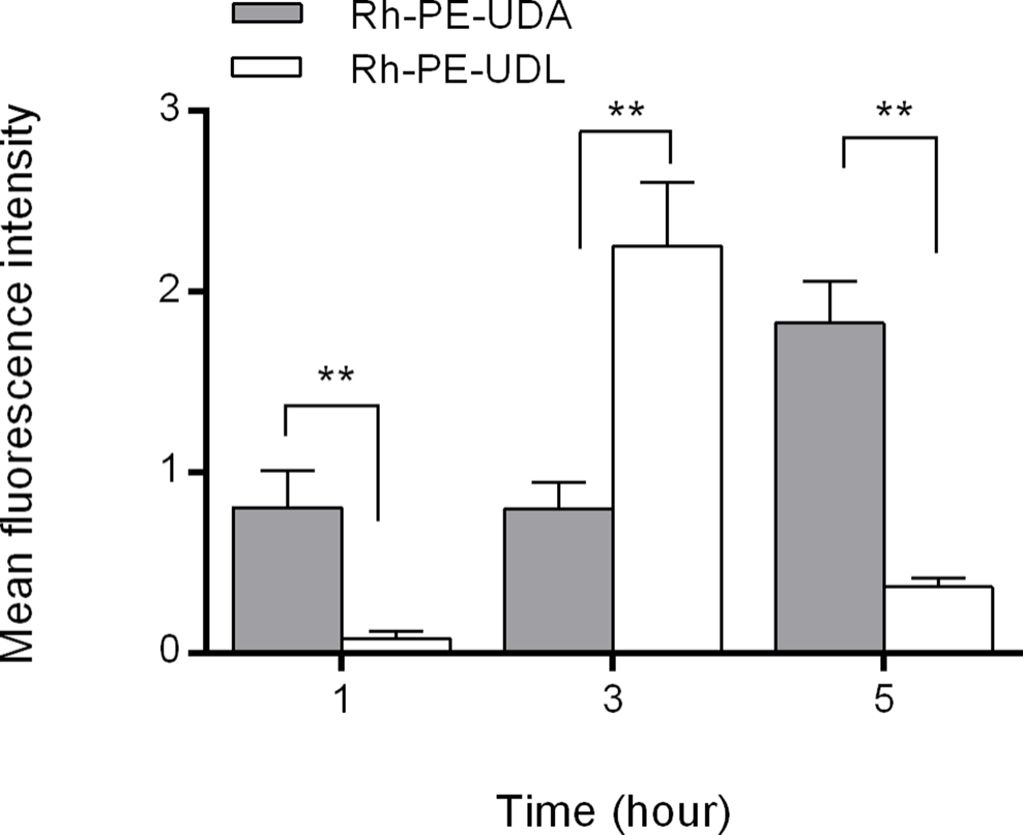
Uptake of Rhodamine–PE labeled UDA and Rhodamine–PE labeled UDL by BMDC, determined by flow cytometry. Values represent mean ± SD, ** denotes p < 0.01

In Fig 5, the uptake of empty nanovesicles by BMDC is shown; a nearly eightfold higher uptake of UDA compared to UDL is observed.

Fluorescently labeled UDA or UDL were incubated 1, 3 or 5 h with BMCD at 37°C. The cells fluorescence resulting from internalized nanovesicles was quantified by flow cytometry.

### Immunization

**Fig 6:**
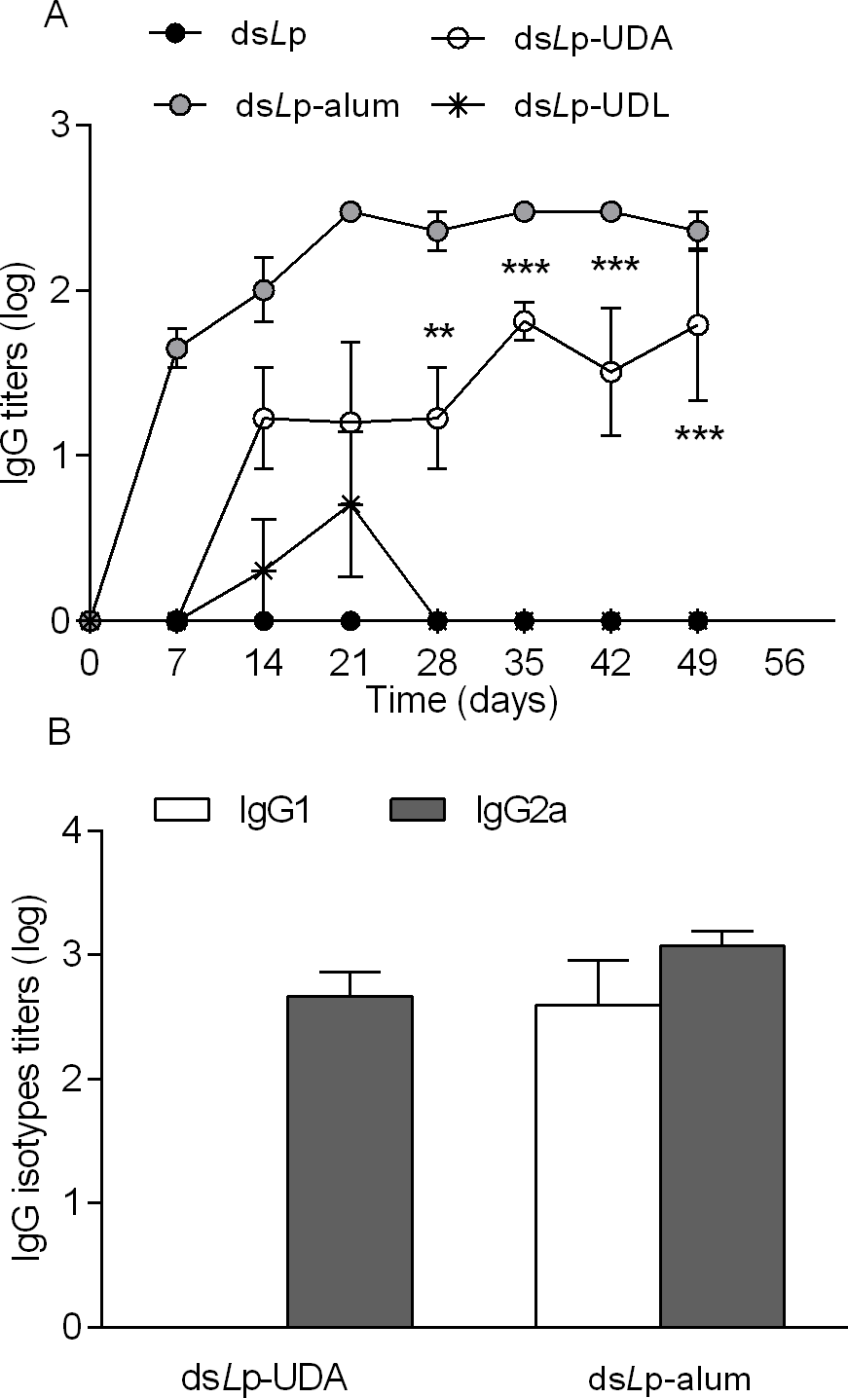
Serum IgG titers after topical application of ds*L*p-nanovesicles and intramuscular application of ds*L*p adsorbed in alum. (A) IgG isotypes. (B) Values represent mean ± SD. ** denotes p < 0.01, *** denotes p < 0.001 vs ds*L*p-UDL and ds*L*p.

The humoral immune response generated after topical weekly applications of ds*L*p-nanovesicles was compared to that generated after topical ds*L*p and by i.m. alum adsorbed ds*L*p, as a positive control of ds*L*p immunogenicity. The titers rose by topical ds*L*p-UDA were 1 log higher than those of ds*L*p-UDL. The response remained constant up to the day 49, while that of ds*L*p-UDL faded rapidly within 3 weeks (Fig 6A). The topical application of ds*L*p did not raise measurable IgG titers. None of the topical administrations induced IgA titers in salivary washes.

In mice, IgG2a produced from Th1 cells indicates cell-mediated immunity, and IgG1 produced from Th2 cells indicates humoral immunity. Therefore, the balance between Th1 and Th2 cells (Th1/Th2 ratio) can be represented as the IgG2a/IgG1 ratio. The isotype analysis of samples taken the 49 day showed that i.m alum adsorbed ds*L*p raised a mixed isotype profile with IgG1/IgG2a ~1. In contrast, ds*L*p-UDA induced preferentially the IgG2a isotype, suggesting a Th1 polarized response (Fig 6B).

## Discussion

**Fig 7:**
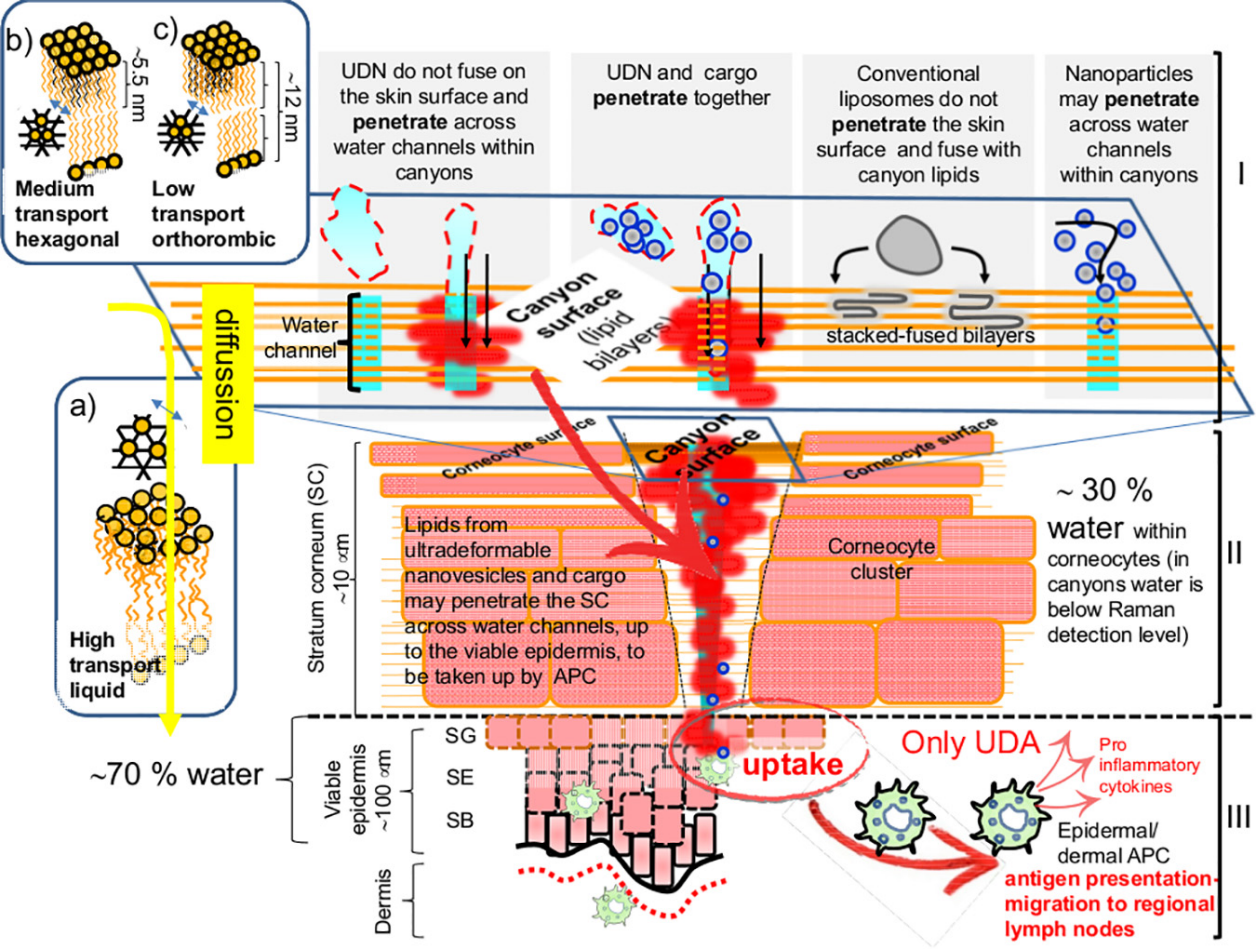
**Scheme depicting the main structural sections of the skin: *stratum corneum* (SC), viable epidermis and dermis, and barriers to permeation-penetration (not a scale):** The diffusive pathway across the lipids of the SC is mediated by disordered bilayers, represented by the X-ray diffraction pattern corresponding to the lateral packing in liquid phase of bilayers shown in a) (distance between planes ∼0.46 nm). Bilayers b) and c) with more organized lateral packing (distance between planes 0.41 and 0.41–0.37 nm, respectively), not involved in diffusion across the skin. The interaction between ultradeformable nanovesicles (UDN), conventional liposomes, hydrophilic solutes and lipids at the SC surface is also represented. Ultradeformable nanovesicles and hydrophilic solutes penetrate across hydrophilic leads in the canyons in-between corneocyte clusters. A scheme of ultradeformable nanovesicles and associated cargo crossing an hydrophilic channel in the SC. The colloidal structure is lost below the surface. The associated cargo penetrates along with the lipid bilayers. No endocytic uptake occurs at I and II levels since the SC is made of dead corneocytes. Below 10 μm depth, endocytic uptake of material penetrating across hydrophilic channels accessing the viable epidermis may occur. Epidermis: *stratum basale* (SB), *stratum spinosum* (SS), *stratum granulosum* (SG). (Dermo epidermal basal membrane is represented as black line and the reticular capillary plexus in the dermis as red dots)

The success of needle free vaccination largely depends on counting on carriers ultradeformable enough to efficiently penetrate the intact *stratum corneum* to bring their payload across the skin. In a previous work, we found that UDA carrying ovalbumin (ova, a 42.7 KDa, 6 nm diameter globular protein) (ova-UDA) raised an IgG2a-biased anti-ova systemic response ten folds higher than ova-UDL, after topical application on Balb/c mice [19]. The immune response to topical stimulus was enabled by the ultradeformable nanovesicles delivering ova across and beyond the *stratum corneum*, presumably through the lipid canyons that separate keratinocyte clusters of the skin upper layers [[47]. Such canyons occasionally extend down to depths comparable to that of the dermal–epidermal junction below the flat surface regions in porcine and human skin [48, 49].Upon topical non-occlusive application, the fluorescence of Alexa Fluor 647 labeled ova within UDL or UDA, allows to indirectly estimate a rough canyons thickness of 50–100 μm [50]. This is in good agreement with the 10–25 μm width canyons separating keratinocyte clusters of 100–250 μm diameter, recently assessed by stimulated Raman scattering microscopy [[50]. Canyons, despite of being wide enough so as to allow the passage of 100 nm diameter nanovesicles, are filled with lipids enclosing water nanochannels, that nanovesicles are thought to penetrate [51]. However, these extremely thin pathways [48, 52]. scarcely distributed across the canyons, together with the impaired material diffusion through the lipid filling organized in orthorhombic lateral packing lamellas [53], and the intercellular unions (corneo-desmosomes in *stratum corneum*, tight junctions in upper viable epidermis) [54], may constitute a physical constraint to the penetration of bulky structures carried by UDL or UDA [49] (Fig 7).

An interesting example is constituted by aqueous suspension of Quantum Dots (QDots, pegylated CdSe/ZnS core/shell 12 nm x 6 nm ellipsoidal bulky stiff nanoparticles), that upon topical application do penetrate the skin up to the viable epidermis. When loaded within ultradeformable nanovesicles however, the QDots remain stacked at the first layers of the *stratum corneum* and do not penetrate [55]. Such observation challenges the notion of needing ultradeformable nanovesicles to grant the penetration of particulate cargo. It suggests also that factors such as size, shape and even stiffness of particulate cargo may account for its penetration, beyond the simple association to ultradeformable nanovesicles (Fig 7). According to this view, the ds*L*p used in this work, of size in the range of a QD (nearly 3 folds higher than ovalbumin), were good candidates to stack on the first *stratum corneum* layers despite of loaded within ultradeformable nanovesicles. Indeed, such phenomenon was suspected while estimating bilayer deformability at the first steps of ds*L*p-nanovesicles structural characterization. When the deformability (*D*, a parameter inversely proportional to the Young modulus (E)) of ds*L*p- nanovesicles was measured by the Van der Bergh method, we found it lower than that of empty ones. On the contrary, the deformability of ds*L*p-nanovesicles determined by AFM was similar to that of empty ones. This apparent discrepancy would obey to a stacking of the oversized (~ 25 nm) ds*L*p mixed micelles during extrusion, which caused a reduced phospholipid flux. The calculated *D* would result from a steric hindrance caused by packing constraints of cargo and not from reduced bilayer deformability. Since the second method measures the cantilever deflection on surface patches of individual nanovesicles, the AFM is thus a more realistic determination of nanovesicles deformability carrying oversized cargoes. As indicated above, the physical constraint imposed by ds*L*p to nanovesicles extruded across 50 nm filter pores, suggested that *in vivo* the ds*L*p- ultradeformable nanovesicles could end up stacked within the *stratum corneum* the same as QD-UDL, thus spoiling the topical immunization. In our experimental setting however, after dropping a weekly dosage of ds*L*p-nanovesicles on the intact skin of Balb/c mice, ds*L*p and ds*L*p-UDL failed at inducing systemic IgG titers, but ds*L*p-UDA was the only topical formulation that successfully raised a sustained systemic antigen specific response. Despite of its low protein/lipid ratio (3.5% vs 12.5% for ova-UDA), ds*L*p-UDA induced 1 log lower serum titers than i.m. ds*L*p-alum adsorbed, but was tenfold more immunogenic than ova-UDA (that rendered 2 log lower serum titers than i.m. ova-alum adsorbed [19]). Topical ds*L*p-UDA induced IgG2a only (which in mice suggests a Th1 biased response, as required for vaccination against *Leishmania*) [6], whereas the response to i.m. ds*L*p alum-adsorbed was a mixed IgG isotype.

As previously determined in [56]., J774A1 macrophages capture empty UDA much more extensively than UDL. UDA in other words is a more efficient carrier for material delivery to phagocytic cells than UDL. Here UDA and UDL showed to be were non immunogenic, since no pro inflammatory cytokines were detected in macrophages supernatants; instead, macrophages responded only to ds*L*p and ds*L*p-nanovesicles. The immune responses to ds*L*p-UDL, however were of low intensity and faded soon. Ds*L*p-UDA was the only capable of inducing an immediate secretion of IL-6, IL-12p40 and TNF-α, followed by high TNF-α and IL-1β titers in J774A1 cells. The response was aroused with lower amounts of proteins (28 μg/ml ds*L*p as ds*L*p-UDA vs 50 μg/ml ds*L*p alone), suggesting that UDA may enhance the immunogenicity of ds*L*p.

In particular, TNF-α level raised by ds*L*p-UDA was not only higher than the induced by ds*L*p alone, but also by the classical TLR4 ligand LPS (the source of the potent MPLA, a well-known immunostimulatory adjuvant present in commercial emulsions such as AS04). Interestingly, ds*L*p-UDA was the only stimulus that induced a remarkable elevation of IL-1β. Similar to TNF-α, IL-1β is a major pro-inflammatory cytokine considered as an alarm signal secreted by macrophages. IL-1β initiates and propagates inflammation by inducing the expression of adhesion molecules on endothelial cells and leukocytes [57, 58]. This cytokine was reported to be involved in a protective immune response against *Leishmania spp*. IL-1β affects the pathogenicity of leishmania by generating an inflammatory response in afflicted tissues and by modulating adaptive T cell mediated immune responses, which act to limit parasite dissemination [59, 60]. Substantive experimental evidence suggests that the MyD88-dependent Toll-like receptor (TLR) signalling, a classical recognition pathway for macrophage activation, is bypassed by the immunologically silent *Leishmania* spp. [61–66]. Instead, IL-1β has recently been described as initiator of the protective immune response. In particular, the NLRP3 (a member of the Nod-like receptor (NLR) family that senses microbes and cell damage) mediated activation of the inflammasome and subsequent induction of IL-1β signalling, was found to play a key role in host resistance to MCL [67]. The inflammasome are multimeric complexes of proteins, assembled in the host cell cytoplasm in response to specific stress signals or contamination of the cytoplasm by microbial molecules. The canonical inflammasomes are composed of at least three main components: an inflammatory caspase (caspase-1, caspase-11), an adapter molecule (such as ASC), and a sensor protein (such as the NLR family members NLRP1, NLRP3, NLRP12; also NAIP1, NAIP2, NAIP5, or AIM2 [68, 69]. Once activated, caspase-1 induces processing and secretion of IL-1β, which is transcriptionally regulated when microbial components are sensed by pattern recognition receptors [70]. Notably, the activation of the inflammasome leads to autonomous macrophage mechanisms that culminate with the restriction of intracellular parasite replication. These processes involve the regulation of IFN-γ and processing of IL-1β, which facilitates the expression of NOS2, an enzyme that is required for NO-mediated restriction of *Leishmania* replication in macrophages [71–73]. The fact that IL-1β signalling is crucial for the determination of the severity of disease in humans, underscores an important role for the inflammasome-dependent restriction of *Leishmania spp*. replication [74, 75].

Unpublished research from our laboratory [76] suggests that one or more archaeolipids present in the total polar lipids of UDA-presumably the main component PGP Me- are ligand of the Scavenger Receptor Class A I/II (SRAI/II). The SRAI/II is a pattern recognition receptor mainly expressed by macrophages, vascular smooth muscle cells and endothelial cells, primarily involved in endocytic uptake of particulate material (lysosomal enzymes, oxidised acetylated lipoproteins, LPS, bacteria) [77]. Since UDA is pronouncedly captured by macrophages upon SRAI/II recognition, the amount of ds*L*p antigens available to interact with NLRP3– a cytoplasmic receptor- would be higher if delivered as ds*L*p-UDA, than as ds*L*p alone or ds*L*p-UDL. This may account for the increase of IL-1β induced on J774A1 cells by ds*L*p-UDA.

On the other hand, the expression of SR A by BMCD is highly dependent on the isolation protocol employed [78, 79]. BMDC were also reported to display NLRP3 mediated inflammasome activation in front to several stimuli [80, 81]. If well the expression of SRA was not screened here, the BMDC have the potential to react similar to macrophages in front to UDA/ds*L*p-UDA. An extensive uptake of UDA however, was the only response recorded by BMDC. Less likely, a concomitant production of pro inflammatory cytokines to ds*L*p-UDA may have occurred in a temporal pattern different to that recorded at 14 or 48 h. These findings suggest the cells involved in an *in vitro* primary response to ds*L*p-UDA were the macrophages. *In vivo*, the proteins in ds*L*p alone were reported to induce IL-12 cytokines [49]. Overall, this first approach showed that the topical Balb/c mice immunization with ds*L*p-UDA generated a Th1 biased response, as determined by the serum isotypes of ds*L*p-specific IgG. Further insights are needed to find out if the high UDA-mediated ds*L*p delivery that led to macrophages secretion of IL-1β, may contribute to an *in vivo* lethal response to *leishmania* parasites.
